# The impact of patient and public involvement on COVID-19 immunology research: experiences from the UK Coronavirus Immunology Consortium

**DOI:** 10.1186/s40900-023-00446-1

**Published:** 2023-05-22

**Authors:** Erika Neves Aquino, Paul Moss, Mo Hafeez, Robert Jasper, Tony Kelly, Lynn Laidlaw, Vivienne Wilkes

**Affiliations:** 1 British Society for Immunology, 9 Appold Street, London, EC2A 2AP UK; 2 Institute of Immunology and Immunotherapy, University of Birmingham, Birmingham, B15 2TT UK; 3 Greater Manchester, UK; 4 West Midlands, UK; 5 Birmingham, UK; 6 Clackmannanshire, UK; 7 East Yorkshire, UK

**Keywords:** Immunology, COVID-19, PPI, Patient and public involvement, Public contributors, Basic science, Laboratory-based science, Measuring impact, Impact

## Abstract

**Background:**

Patient and Public Involvement (PPI) in clinical trial research is recognised as relevant but the active involvement of patients and the public in basic science or laboratory-based research is seen as more challenging and not often reported. PPI within the UK Coronavirus Immunology Consortium (UK-CIC), a translational research project aimed at tackling some of the key questions about the immune system’s response to SARS-CoV-2, is an example of overcoming negative perceptions and obstacles. Given the widespread impact of COVID-19, it was important to consider the impact of UK-CIC research on patients and the public throughout, and the PPI panel were an integral part of the consortium.

**Findings:**

Building in funding for a PPI panel to value involvement and ensuring effective expert administrative support and management of PPI were crucial to success. Facilitating relationships and quality interactions between public contributors and researchers required time and commitment to the project from all parties. Through creating a platform and open space to explore diverse views and a wide range of perspectives, PPI was able to influence researchers’ ways of thinking about their research and impact future research questions about COVID-19 immunology. Moreover, there was long-term impact from the involvement of the PPI panel in COVID-19 research and their value was reflected in invitations to contribute to additional immunology projects.

**Conclusion:**

The ability to conduct meaningful PPI with basic immunology research has been shown possible through the UK-CIC in the context of the fast-moving COVID-19 pandemic. The UK-CIC project has laid the foundations for PPI in immunology and this should now be built upon for the advantage of future basic scientific research; PPI can impact greatly on laboratory-based research when given the opportunity to do so.

**Supplementary Information:**

The online version contains supplementary material available at 10.1186/s40900-023-00446-1.

## Abstract (plain language summary)

The UK Coronavirus Immunology Consortium (UK-CIC) was established to address key questions about the immune system’s response to SARS-CoV-2, the virus which caused the COVID-19 pandemic, to benefit patient and public health at pace. The project brought together immunology laboratory-based research with the lived experience of patient and public contributors to ensure that the priorities for these groups and impacts of the research on the wider public were considered throughout. A Patient and Public Involvement (PPI) panel was set up to meet regularly with the scientists to provide a unique perspective, ask pertinent questions about the implications of the research, and discuss what was important to the public and patient groups. This paper examines the practical resources, support, and relationship building necessary to embed PPI in basic scientific research as well as how this can be achieved within urgent, critical research conducted during a pandemic situation. It explores the successes, impacts and legacy of involvement for researchers, the research, and importantly, the patient and public contributors. By sharing experiences from UK-CIC to help dispel any existing misconceptions that PPI can act as a hindrance, this paper proposes greater encouragement of PPI in all basic science research.

**Supplementary Information:**

The online version contains supplementary material available at 10.1186/s40900-023-00446-1.

## Background

The UK Coronavirus Immunology Consortium (UK-CIC) [1] was a UK-wide study aimed at tackling some of the key questions about the immune system’s response to SARS-CoV-2 for the improvement of patient care and the development of better diagnostics, treatments and vaccines for COVID-19. The UK-CIC was initiated in August 2020, funded for 12 months by UK Research and Innovation (UKRI) and the National Institute for Health Research (NIHR), and supported by the British Society for Immunology (BSI). The immune system is extremely complex, and so to make rapid and effective progress towards understanding the immunology of COVID-19, a cohesive and nationally coordinated approach was required. The UK-CIC brought together the expertise and specialist resources of 20 centres and over 200 researchers around the UK to deliver real benefits to patient and public health at pace.

Given the widespread impact of COVID-19, the views of patients and the public were central to the success of UK-CIC. It was important to consider the impact of UK-CIC research on patients and the public throughout, and the PPI panel were an integral part of the consortium.

Involving patients and the public in research is described by the NIHR as doing things ‘with’ or ‘by’ people, rather than ‘for’ or ‘to’ them [2] and there is growing evidence of the positive impact that meaningful involvement can have in medical research [3]. Whereas PPI in clinical trial research is increasing and perceived to be relevant, the active involvement of patients and the public in basic science or laboratory-based research is perceived as more challenging and not often reported [4]. PPI in research is vital to ensure studies are relevant, effective and measure the outcomes meaningful to the people affected, so improving the quality of research. Despite this, barriers to involving people in complex fundamental science still remain [5]. This paper has been written within this context and highlights how PPI within the UK-CIC is an example of overcoming those obstacles and negative perceptions. It also provides practical suggestions on how to bridge the gap between basic research and the lived experiences of patients and the public.

**Fig. 1:**
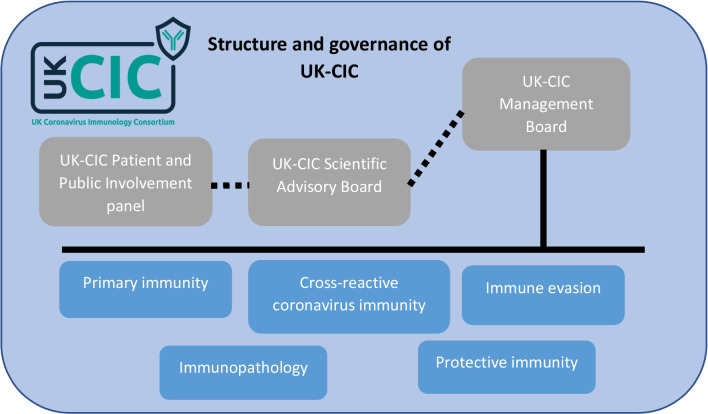
Structure and governance of UK-CIC

The UK-CIC governance structure consisted of a Management Board to directly oversee the project delivery and an independent Scientific Advisory Board, which the PPI panel feed into (Fig. 1). Further details of these Boards are given in Additional file 1.

A subgroup of five members from the ten-member PPI panel (Mo Hafeez, Robert Jasper, Tony Kelly, Lynn Laidlaw and Vivienne Wilkes) along with Erika Aquino, the Public Engagement Manager at the BSI who led the PPI work within UK-CIC, have co-authored this manuscript following guidelines and criteria as outlined by Richards et al. (2020) [6]. All other members of the PPI panel were also given the opportunity to add their perspectives and significantly contributed to the content of this paper. We thank them very much for their input.

## Main text

### Setting up and supporting PPI in the project

Two public contributors, Robert Jasper and Tony Kelly, were invited to join the project by the Principal Investigator during the grant application stage. Understanding the importance of PPI, Professor Paul Moss reached out to the local NIHR Birmingham Biomedical Research Centre for suitable public contributors and both Robert and Tony have lived experience and are advocates for people with health conditions that make them more vulnerable to COVID-19, as well as having previous research and PPI experience. At this stage, involving, and crucially funding, the BSI to bring specialist PPI knowledge and management to the project allowed the PPI function to flourish and facilitated the successful involvement of patients and the public.

Robert and Tony helped the BSI to set up the PPI panel. To define the nature and scope of involvement, a Terms of Reference (see Additional file 2) was created using a template from the NIHR INVOLVE website [7, 8] and, incorporating feedback from Robert and Tony, was used to recruit additional members to the panel. Due to the tight time pressures of the project, the panel had to be recruited quickly to start immediately and proceed at a fast pace. The BSI recruited the panel by contacting relevant medical research charities and organisations that had established and experienced PPI groups to seek expression of interest from diverse and inclusive communities. A description of the panel’s purpose, responsibilities, ways of working and remuneration was circulated to extensive networks. Those recruited needed access to the internet, an accessible device to attend virtual meetings via Zoom and an email address for communications.

Members of the PPI panel were recruited based on the UK-CIC public health research priorities and included COVID-19 survivors; people from groups deemed clinically extremely vulnerable such as people who have diabetes or cardiovascular conditions or who take immunosuppressive therapies; people from minority ethnic backgrounds; people aged 70-years-old or over and parents with children who are clinically vulnerable. The panel was balanced with regard to gender and members were sought from around the four nations to represent the nature of a UK-wide consortium. Attention was given to recruiting people with relevant experiential knowledge, to ensure the PPI panel input would be most useful and had increased potential for impact [9]. Over 30 people expressed interest in joining the PPI panel and the BSI held initial virtual one-to-one meetings with 25 individuals to further understand their experiences and motivations for joining the panel and as a selection process. Great care was then taken to select a group who would work well together and complement each other.

**Fig. 2:**
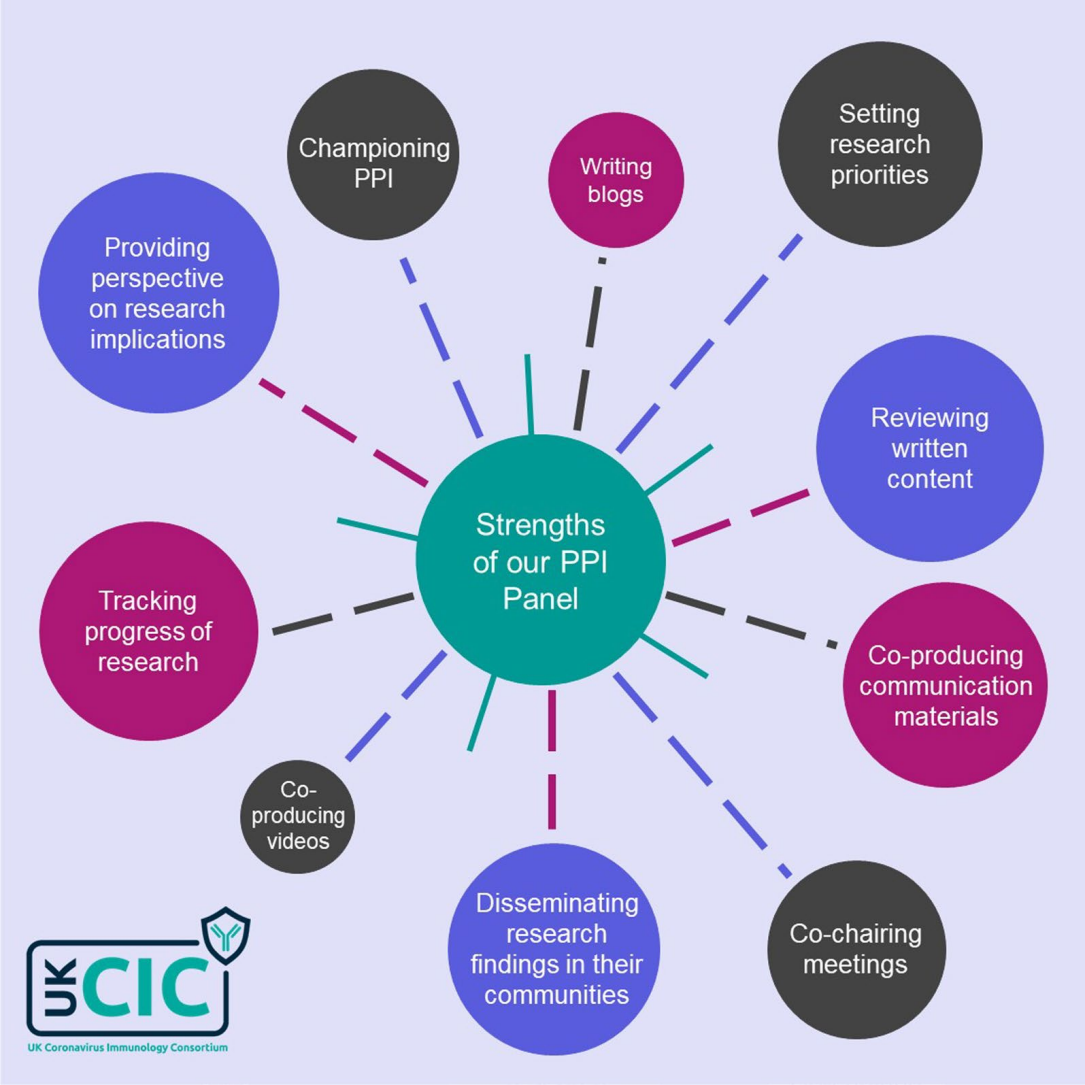
Strengths of UK-CIC PPI panel members

The final PPI panel was made up of ten members with diverse backgrounds and experiences, and a broad set of knowledge and skills (Fig. 2), which are detailed in the self-described profiles hosted on the UK-CIC website [10] and in Additional file 1. A wide range of patient and public groups were successfully represented through the PPI panel and members regularly consulted their communities and inputted collective views into discussions. At the time of recruitment in 2020, everyone was affected by COVID-19 and the project endeavoured to involve a broad range of people from the general population but it was acknowledged that achieving genuine representation of a whole population was unfeasible and not the aim. Nonetheless, multiple views and perspectives were embraced in an inclusive and respectful environment and ensured the panel members became a powerful voice at the table [11].

*My highlight of PPI in UK-CIC has been working with the most diverse group of people I’ve come across, with so many great qualities. I’ve enjoyed learning about the different lived experiences of our panel members and have gained an increased awareness of what is important for those patients. –UK-CIC PPI representative and Advisory Board member*

The PPI panel met monthly on Zoom for the duration of the project. Each meeting was co-chaired by Erika Aquino from the BSI and one member of the panel, rotated around the group as was suggested in early discussions by the group themselves. The BSI led on preparation of the meeting agenda and the panel member opening the meeting with introductions and working to keep the conversation focused and engaging, taking questions and managing timekeeping. This allowed everyone a chance to gain these essential skills and shared the workload between the group. The BSI briefed each co-chair prior to the panel meeting and discussed the agenda and logistics, enabling the PPI panel members to input on how the meeting would run.

At each meeting, different UK-CIC researchers were invited to give a short presentation to the group and there was plenty of time factored in for questions, two-way conversations, and discussions with the panel. PowerPoint presentations were shared in advance of the meetings, which panel members, especially those with accessibility issues, found helpful and allowed them to keep a record of presentations. Every meeting also covered feedback from Robert and Tony from the previous Advisory Board meeting meaning they could raise any topics that needed PPI input, as well as updates from the UK-CIC co-chairs, who alternated attendance. Panel members were offered an honorarium of £45 for each meeting as recognition of preparation time, reading of papers in advance and playing an active, constructive and co-operative role in meetings. This was within budget restrictions and based on NIHR guidance for involvement in an approximately two-hour activity requiring some preparation [12]. Panel members verbally complimented the BSI’s prompt payment during meetings and expressed how this made their input feel valued, which is recognised as important for authentic involvement [13].

After every meeting, a follow up email was sent by the BSI with any materials or links that had been shared in the Zoom chat and the link to the Padlet, which is an anonymous online feedback tool for comments around what went well in the meeting as well as what could be changed for the next meeting. A reminder was also included that panel members could contact the BSI confidentially by email or phone call outside of the meeting with any comments or concerns if they were uncomfortable sharing in the group.

Establishing the PPI panel assisted the project to remain fluid, meeting the changing dynamics of the pandemic. When wider understanding of COVID-19 increased and vaccine rollout began, the research undertaken by the UK-CIC progressed and the responsibilities of the PPI panel developed. The members of the panel reported feeling more confident and comfortable in asking pertinent questions directly to the scientists, placing the research in the context of the concerns and needs of the public, as well as putting forward questions they felt were important to be addressed. Towards the end of the project, the PPI panel had built an influential reputation amongst the UK-CIC researchers and funding bodies, and they were approached to participate in other external activities. Involvement with UK-CIC allowed the public contributors' networks to grow and, anecdotally, panel members reported that they were invited and approached by other organisations who specifically asked them because they had heard of the great work achieved in the UK-CIC.

### PPI activities

Alongside the monthly meetings, the PPI panel were involved in other UK-CIC public engagement activities. The panel worked closely with Erika Aquino, Public Engagement Manager, and Gabriela De Sousa, Research Communications Officer for UK-CIC, to ensure all external communications were accessible in format and language to increase public understanding of how fundamental immunology research can lead to beneficial diagnostics and treatments for people affected by SARS-CoV-2. The panel helped develop the UK-CIC website’s ‘for the public’ section [14], with ideas and suggestions on content and layout to make the page practical, user friendly, and easy to read and navigate. The website was also clear about the importance of PPI within UK-CIC and each panel member had a profile on a dedicated PPI page [10], which sits prominently in the ‘about us’ section. Two panel members, Mo Hafeez and Lynn Laidlaw, were also featured in short inspiring videos [15] to explain PPI and how it focuses research efforts to deliver real benefits and change to patients and the public. Additionally, the PPI panel reviewed all public summaries [16] of the published research, providing feedback and critiques on the writing, for which they were offered supplementary remuneration for their time.

Through being involved in these public engagement activities, panel members were delighted to be able to help their wider communities to better understand the pandemic by sharing information and resources. The panel commended that the BSI worked with members who have accessibility requirements to ensure the website content and shared documents were accessible to all.

*PPI with UK-CIC has been brilliant for the visually impaired community as it has led to increased accessibility of information, which is so important. All too often we are excluded from information because of lack of accessibility; this was especially true at the start of the COVID-19 pandemic. The UK-CIC website meant that we could independently digest information and that is something UK-CIC should be very proud of. UK-CIC has made science less scary and within reach for our community.*

–UK-CIC PPI representative

**Fig. 3:**
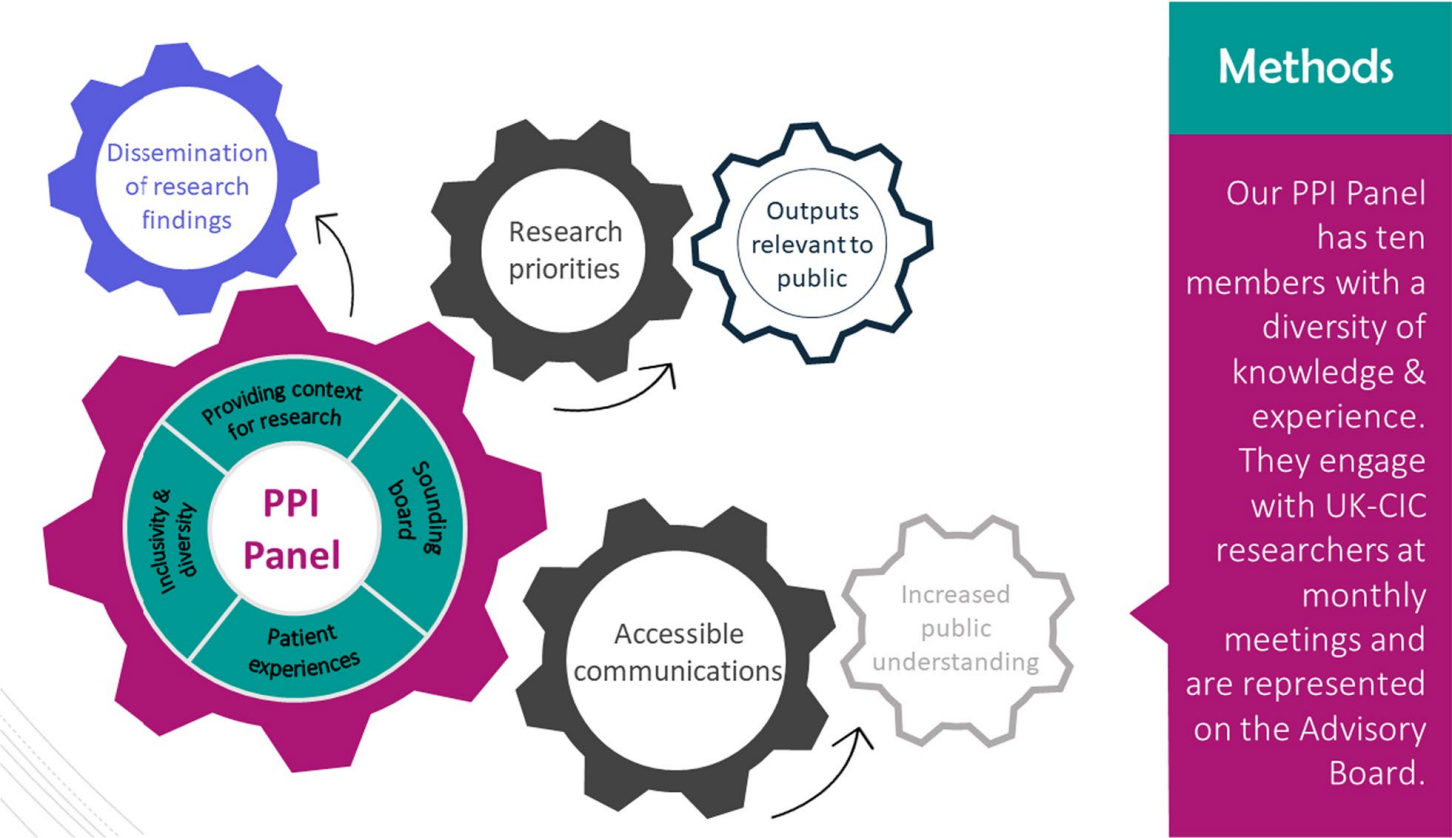
Methods section of the PPI poster at the UK-CIC virtual conference

**Fig. 4:**
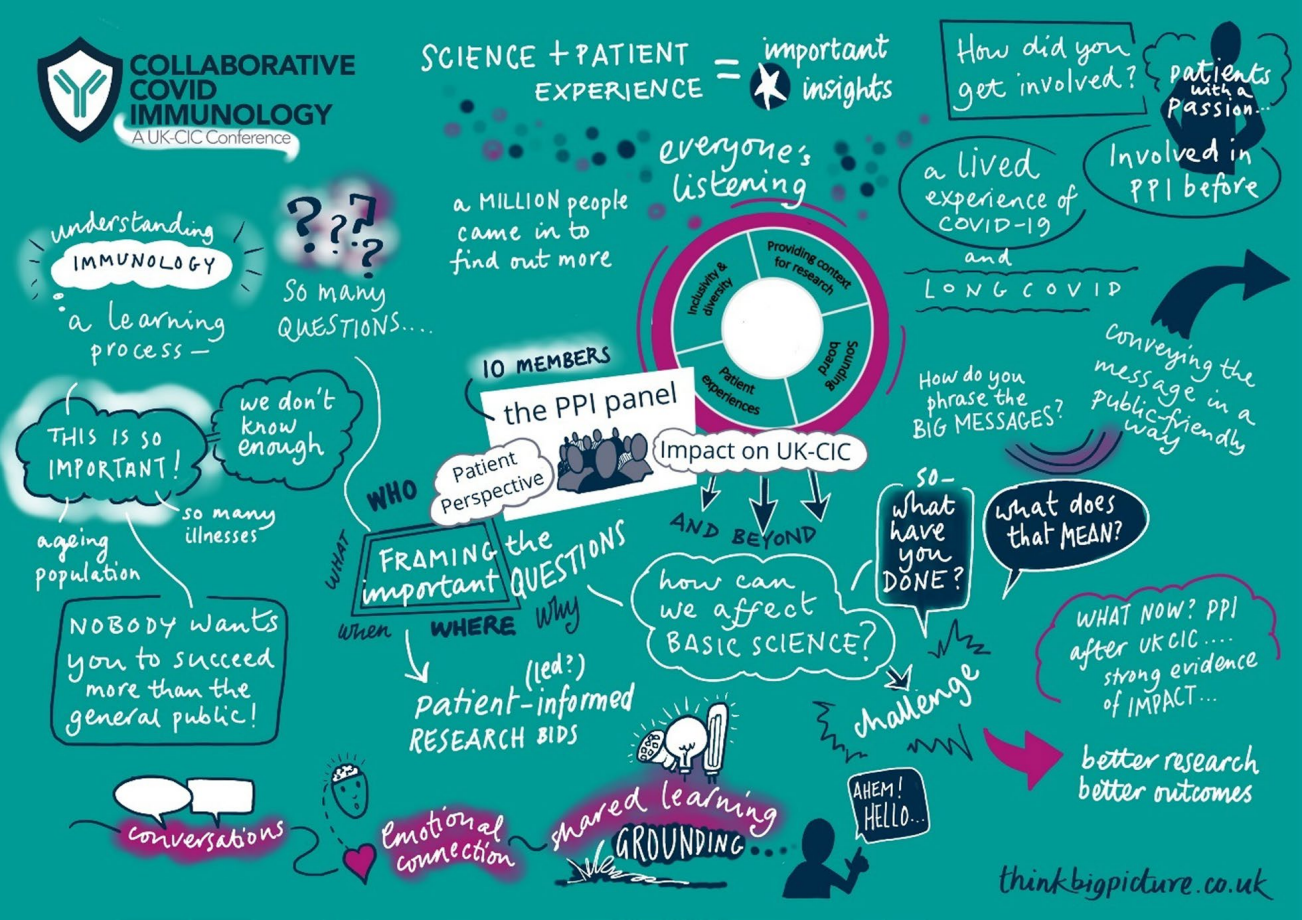
Illustration to capture one of the PPI 'chat room' discussion at the UK-CIC virtual conference

Furthermore, PPI was showcased at the UK-CIC scientific virtual conference 'Collaborative Covid Immunology', held on 28–29 April 2021. Although aimed at an academic audience, PPI was a visible theme throughout, and a recommendation of the PPI panel meant all scientific abstracts had to include a public summary to make the research more accessible. A subgroup of the PPI panel suggested, created and presented a poster about the impact PPI had on UK-CIC (Fig. 3) which generated conversations about the practicalities of meaningful involvement. On both days of the conference, there was a PPI ‘chat room’ where PPI representatives and researchers could meet to have focused discussions on the importance and impact of PPI and hear from the patient perspectives on UK-CIC research. These sessions were well attended and created a space for interactive and thought-provoking dialogues, which were captured by an illustrator and can be seen in Fig. 4. Prominent in the conference programme and ahead of the plenary session, PPI representative Tony Kelly gave a powerful speech about the value of PPI in research and the importance of genuine two-way partnerships, which was seen by 209 delegates and noted as a highlight of the conference by many in the formal feedback survey and personal communications. The video recording was also available to watch on the UK-CIC website [10].

*As a researcher I find I can learn a lot from PPI; there’s excellent work happening here which I hope will continue. I really enjoyed Tony's power-packed 5 min—really important message! –UK-CIC conference delegate feedback*

Outside of the conference itself, the PPI panel helped develop several activities to engage with the public about the work of the consortium. Short, engaging, public-friendly profiles [17] of UK-CIC researchers were created for social media, which came from conversations with the PPI panel about the public image of researchers and the need to showcase scientists in a human light. The PPI panel were passionate about communicating the scientific achievements of UK-CIC to a wide audience and so two free public webinars [18] were held about ‘COVID-19 and your immune system’ and ‘COVID-19, vaccines and protective immunity’. UK-CIC researchers presented their key findings and took questions from the audience, with both events being co-chaired by a PPI panel member. These webinars were hugely successful with over 600 live attendees and the recordings are also available to watch on the UK-CIC website. PPI panel members were also keen to engage with the public on different platforms and so there was a UK-CIC Reddit ‘Ask Me Anything’ [19], where researchers answered public questions about COVID-19 immunology. The researchers could interact live for two hours and the content that had the most ‘upvotes’ included discussions around the length of time immunity to COVID-19 might last for and what the biggest unknowns about COVID-19 were. This activity had brilliant engagement and after 24 h the post had received 605 comments.

### PPI influence and successes

Through meeting with the PPI panel, researchers were provided with a platform for two-way dialogue and a sounding board to explore their research priorities. Valuing individual experiences and exploring different perspectives on research findings ensured that the outputs of UK-CIC were relevant to the public and aligned with patient interests.

*PPI helps to make sure we think through the ultimate application of our research for the public. This can initially be challenging to address and so it may even make scientists feel a little defensive. But like all things in life, after you apply yourself, you learn from it and it makes you stronger.*

–UK-CIC Principal Investigator

**Fig. 5:**
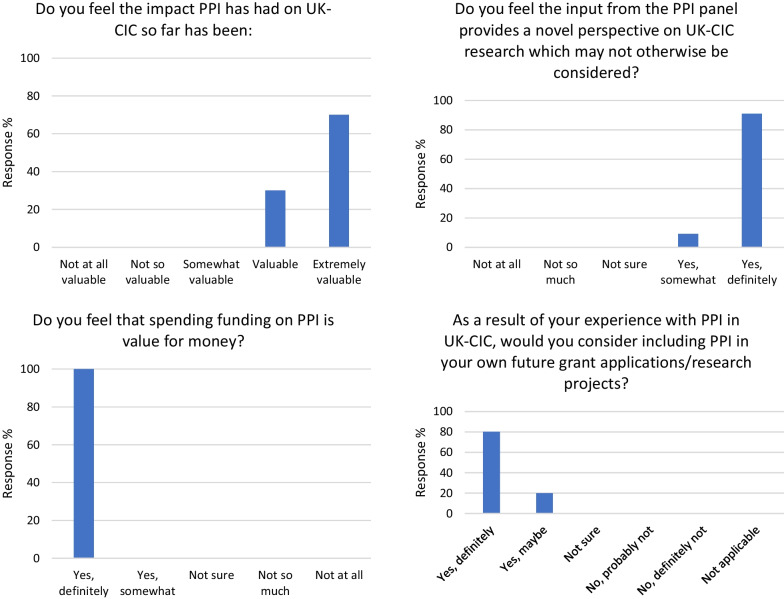
Results from Advisory Board poll

The Advisory Board regularly provided anonymous feedback on PPI, by using the poll function on Zoom during their own meetings. This was an easy and quick way to capture feedback and allowed for honest responses. At the January 2021 Advisory Board meeting, 70% said that the feedback from the PPI panel had changed their views towards the research agenda of UK-CIC and this positive response increased to 90% in June 2021, showing the progression of PPI and how the panel’s feedback became more significant over the course of the project. It was found that 100% of the Advisory Board agreed that: the impact PPI had on UK-CIC had been valuable or extremely valuable, the input from the PPI panel provided a novel perspective on UK-CIC research which may not otherwise be considered, spending funding on PPI was value for money and as a result of their experience with PPI in UK-CIC they would consider including PPI in their own future research projects (see Fig. 5)

The Management Board, who had a different relationship with the PPI panel, were polled once at the end of the project in June 2021. 83% agreed that the impact PPI had on UK-CIC had been valuable or extremely valuable, 67% felt that the input from the PPI panel provided a novel perspective on UK-CIC research which may not otherwise be considered, 83% said that spending funding on PPI was value for money, and 83% said that as a result of their experience with PPI in UK-CIC they would consider including PPI in future research projects. As well as the positive impact on the UK-CIC leadership team, PPI was able to impact directly on the researchers and their research. Following findings by Staley et al. (2017) [20], all researchers that engaged with the PPI panel were asked to provide feedback on their experiences which helped to understand how involvement could influence researchers’ thinking to have an impact on research. All comments were encouraging, with researchers finding the meetings rewarding, useful, enjoyable, valuable as well as challenging and constructive. Many researchers commented on how welcoming, engaged and knowledgeable the panel was and how it was a pleasure to meet with them.

*I really enjoyed presenting to the PPI panel; they’re very cohesive and critical in the right way. This was my first time at a PPI meeting and, while I was unsure what to expect, as soon as I logged on [to Zoom], I realised the tone was very warm and welcoming and the idea is that they work with us to ensure maximum impact of the UK-CIC studies. It’s a fantastic resource to have access to.*

–UK-CIC researcher

*I found the PPI panel stimulating and enlightening and without a doubt improves the quality of our research.*

–UK-CIC researcher

The format of the meetings, in which most of the time was given for open discussions and presentations were only short, allowed the focus to be on the panel’s views and thoughtful, relevant questions. Presenters were also happy to answer additional questions via email after the meetings. The PPI panel made sure that equity, equality, and diversity featured meaningfully at the meetings and that historically underserved communities were placed at the forefront of discussions. The panel were passionate about diversity and inclusion, which was explicitly raised as well as being embedded in the group culture, and everyone strived to create an environment that enabled all communities to be part of the conversation. The PPI panel also promoted and valued the diversity of the participants involved in the research because of the impact COVID-19 had on minority ethnic groups and marginalised communities. Researchers commented that their experience with the panel made them reflect on how they communicate their findings and talk about using patient samples, which needs sensitive and clear language. Additionally, panel feedback suggested some early researcher presentations were too technical and so future presenters were briefed beforehand to minimise jargon and acronyms and fully explain any graphs. The panel later praised the disciplined plain English approach to presentations.

PPI was also an opportunity for researchers to assure people that their basic immunology research was relevant to the public. Realising the value of PPI, some researchers felt motivated to include more patient and public involvement and engagement in their work and left the meetings inspired.

*I’ve been spurred on to have further discussions about engagement and involvement with my department.*

–UK-CIC researcher

We found the biggest impact of PPI was around how the researchers thought about their work and approached their research questions. Often, this impact of involvement is difficult to measure as it can be subjective [21]. However, researchers reported that conversations with the panel helped them to learn from a range of experiential knowledge. For example, an immediate outcome of the PPI meetings was that researchers learnt about the lived experiences of individuals with long COVID, autoimmune diseases and other long-term health issues, which led them to change their thinking about future work to understand patient perspectives and prompted them to seek new collaborations.

Researchers described how they better understood the importance of involving patient and public perspectives from the beginning of a research project and moving forward they would endeavour to embed PPI at the start of future grant applications. One researcher went on to apply for further funding to work on an idea generated from the conversations with the panel.

*The questions we are addressing were deemed important by the panel, but they also had lots of other highly useful suggestions. I found it extremely rewarding and took away 2 pages of notes!*

–UK-CIC researcher

Meeting the panel made researchers consider which questions were most important to the individuals affected and the discussions allowed them the space and time to step back to see the bigger picture.

*The conversations with the panel made it easier for me to think about what we're doing in terms of the individual people it might impact (and what they want) rather than just thinking about populations.*

–UK-CIC researcher

*PPI has bridged the gap between lab and lived experience and UK-CIC has proven that personal experiences have a place within basic scientific projects.*

–UK-CIC PPI representative

As well as the impact on research and researchers, a huge impact of being involved was on the individuals of the panel. The UK-CIC project provided panel members with something meaningful to be part of during the COVID-19 pandemic, which was an emotional and difficult period. However, they were enabled and empowered through being personally involved in efforts to bring the pandemic under control and in gaining new knowledge of basic research, immunology and COVID-19. Panel members felt valued and listened to and that it was a privilege to interact with scientists that were prominent in UK media and as Government advisors at the time. Many members mentioned how much they learnt from each other and how their confidence in PPI had been boosted by their positive experience. Gathering individual qualitative feedback was essential to capture unintended impacts, which are recognised as important motivators for PPI contributors to remain involved in research projects [22].

*Working with UK-CIC has made me proud of PPI. I want to now promote my experience to encourage others to work more with research. I’ve been reading up on the huge amount of information about immunology and have enjoyed learning about the endless research which is happening at UK-CIC.*

–UK-CIC PPI representative

Another success of PPI within UK-CIC was the relationships that formed throughout the project and a great sense of teamwork in everything that was achieved. This success was reflected in a high average 90% attendance at every meeting, and effectively retaining all ten members of the panel for the full duration of the project. Great rapport was built in all directions between the panel members, between the panel and the BSI as well as the UK-CIC leadership team and the individual researchers. Moreover, these relationships were so valued that all the public contributors were keen to remain in touch with the BSI for future opportunities to be involved in immunology research and they were subsequently approached by the UK-CIC Principal Investigator to join another COVID-19 research project, the National Core Studies Immunity programme, because of their capability and effectiveness as a PPI panel. This relationship management required both financial resources and time and was facilitated by the BSI, including Erika Aquino always being accessible and responsive by email and phone as well as the administrative support from Laura Anderson, UK-CIC Administrative Assistant, with scheduling meeting dates, circulating agendas and papers, and capturing minutes.

*Working with the PPI panel has been a privilege and I’ve appreciated seeing the relationships develop between all parties. There’s a mutual respect between the researchers and PPI representatives, which has been the foundations of impactful and meaningful involvement.*

–UK-CIC PPI lead

*It has been wonderful to witness the energy and commitment that the PPI panel has brought to the project.*

–UK-CIC Advisory Board member

### Limitations and lessons learnt

The funding application stage of the project had to move quickly at the start of the pandemic when there were many unknowns about COVID-19 immunology and as such, it was understandable that research priorities were set by the researchers and independent scientists. Recruitment of the PPI panel unfortunately therefore came after funding was awarded and the BSI was appointed to manage the PPI function. Ideally, PPI should be considered before the initiation of a research project and involvement of patients and the public needs to happen from the start when formulating research questions to ensure the research is relevant, appropriate and meets the needs of the people who it is designed for. This was not a unique occurrence during the COVID-19 pandemic, with the Health Research Authority (HRA) reporting a significant drop in PPI in research studies submitted for approval in March – April 2020 [23]. The HRA created a fast-track process for researchers planning urgent COVID-19 research to access PPI support and showed that, with effective collaboration, PPI can be a core part of the way pandemic research is conducted in the UK. This is an important lesson learnt within UK-CIC and many of the researchers, after meeting the PPI panel, mentioned that it would be highly useful to talk to such a group when designing grant applications. Involving patients and the public in this early step of the research process helps to develop research that is important to the public with better understanding of the lived experience of the people who will benefit, as well as being an important sense check for language and terminology used in the application.

Problems associated with digital exclusion were recognised at the point of recruitment but because the project was taking place during the COVID-19 pandemic, this was unavoidable. There are limitations to exclusively meeting online and for future involvement projects, meetings need to be suitable for those people who the research is trying to involve, as discussed by Adeyemi et al. [24]. Panel members were also offered the opportunity to be contacted via phone calls at any stage if they were unable to join online.

The PPI panel established themselves as an integral part of UK-CIC in an evolutionary, as opposed to instantaneous, way. Bringing together academic researchers and PPI contributors to learn from each other helped debunk the myth of ‘us versus them’ in science. Although these groups often work on parallel lines, the PPI panel meetings provided a vital crossroad and proved that working collaboratively rather than in isolation was more effective. As discussed by Staley & Barron (2019), [21] the quality of interactions between PPI contributors and researchers was vital for wider impact on the research culture of UK-CIC. As summarised by the UK-CIC Advisory Board chair below, it took time for the researchers to recognise the important role of the panel, but they did come to see that their involvement supplemented and enriched the work of UK-CIC. As recognised by Boylan et al. (2019) [25], involving public contributors in research requires social support from colleagues and ‘buy in’ from senior staff, which in the UK-CIC was championed by the Advisory Board and leadership team. Like findings from Boylan et al., researchers’ attitudes towards PPI were a key influence on how involvement was embedded in the immunology research culture.

*In the early stages of the project, PPI was met with some reluctance with many researchers having no experience in PPI previously. However, over time the contributions from the PPI panel became more and more appreciated. The researchers really took the importance of PPI to heart, and this change of mindset happened among many UK-CIC researchers.*

–UK-CIC Advisory Board chair

The Advisory and Management Boards felt that PPI in the consortium was very successful overall, but when asked what they felt were barriers that prevented PPI from achieving maximum potential impact within UK-CIC, feedback included lack of knowledge about PPI within UK-CIC. An essential way to improve PPI impact in any research project and something that could have been improved in UK-CIC was to partner with public contributors and researchers with experience to provide training before and during the project, covering what PPI is, why it’s important and how best to do it to develop knowledge and support peer-to-peer learning from sharing experience. Increased awareness of and resources for PPI within UK-CIC would have benefited the project and more researchers could have seized the opportunity to interact with the panel. Over 12 meetings, a total of 17 researchers engaged with the panel and were encouraged to cascade information to their colleagues about the benefits of involvement.

A valuable lesson learnt throughout the project was the importance of evaluating PPI as we went along and including everyone’s perspective through asking all parties for feedback [26]. After each meeting, anonymous feedback was gathered from the panel through Padlet, an online pin board, where people could post comments to questions such as ‘What went well? What did you enjoy?’ and ‘What could be improved? What can be done differently next time?’. Comments and suggestions were taken on board to improve future meetings and the feedback was reviewed with the panel every three months.

The PPI function was assessed and improved iteratively in response to experience, and this was particularly useful to identify any challenges and what had to be adapted to overcome them. Critically, this was helpful when discussions during the meetings diverged away from the remit of UK-CIC research. Understandably, many panel members were concerned about COVID-19 vaccine hesitancy within their communities but there was feedback that these conversations within the meetings lacked focus. Using feedback to address the issue, that challenge was overcome successfully by inviting researchers who worked on the immunology of vaccination to speak to the panel and reiterate the need to understand fundamentally how vaccines work. Through these actions, the panel were able to refocus on the aims of UK-CIC. Furthermore, revisiting the UK-CIC research goals with the panel and exploring how basic immunology research could deliver benefits to public and patient health meant that the relevance of UK-CIC became clearer as the project progressed.

*When I discovered what the research was going to be about, I thought it was purely academic and not related to halting the pandemic, so I didn’t have any great expectations for the PPI. As the year progressed, I understood how the academic research helped understand the virus’ effects on the immune system which contribute to beneficial treatments for patients, proving that UK-CIC research is worthwhile.*

–UK-CIC PPI representative and Advisory Board member

The practicality of being able to record on Zoom for note-taking purposes allowed for more detailed and accurate meeting minutes. The panel commended the comprehensive accounts of the meetings, which allowed Robert and Tony to easily report back to the Advisory Board and for the Advisory and Management Boards to have a clear picture of how PPI was functioning. Additionally, clear and concise communication via email was vital when working remotely to ensure everyone had all information needed to prepare and attend meetings.

All these learnings were applied to a future immunology research project, the National Core Studies Immunity programme, involving the Principal Investigator and PPI panel. Longitudinal impacts from UK-CIC have been seen in this following project, where the conversations started in UK-CIC influenced future research questions. The ability to change researchers’ ways of thinking and guide subsequent research during a pandemic setting is an impact to be proud of. For example, two of the public contributors were invited to be co-applicants on a COVID-19 vaccine student directly because of their work on UK-CIC and influenced the data analysis plans of that project. Furthermore, based on discussions during a UK-CIC PPI panel meeting, a researcher has gone on to successfully apply for funding to understand how effective COVID-19 vaccines are in people with a weakened immune system and three members of the UK-CIC panel were involved in the application writing stage.

## Conclusions

The ability to conduct meaningful PPI with basic immunology research has been shown possible through the UK-CIC in the context of the fast-moving COVID-19 pandemic. Building in funding for a PPI panel, ensuring effective expert administrative support and facilitating relationships between researchers and the PPI panel were crucial to success. Working online allowed the PPI panel to come together efficiently and quickly and removed many accessibility and travel barriers, similar to the positive experience of Jamal et al. [27]. The pandemic context may have increased motivation of all involved due to the urgent and direct concern touching everyone’s lives, which resulted in intense involvement. On the other hand, many people were facing personal challenges during the pandemic, such as home-schooling children or caring for dependents, which will have reduced their availability for involvement and having to move to online engagement will have excluded marginalised groups further as also noted by Sproson et al. [28]. PPI within individual projects is a unique experience but advice from other’s lessons learnt can be useful.

This paper hopes to have helped dispel the existing misconceptions that PPI in basic science is not possible [29] and by sharing best practice it has shown that involving patients and the public can enhance research. The involvement methods implemented within UK-CIC are not claiming to be the only or best approach but instead, this paper seeks to encourage and inspire others. The experiences shared in this paper are evidence that the invaluable contributions from patients and the public from all backgrounds should not be underestimated.

Involvement within UK-CIC aimed to provide patient and public perspectives on the priorities and impacts of the research and this was achieved through regular interactions between the PPI panel and the Principal Investigators, workstream leads and scientists. Pertinent discussions within the context of a changing pandemic were facilitated in a trusted space and everyone’s views were respected and listened to. Involving patients and the public improved outputs and outcomes for UK-CIC and is an example of basic science benefitting positively from PPI.

Scientists must not ignore or overlook the significance and relevance of PPI and instead consider it a mutually beneficial relationship. The UK-CIC project has laid the foundations for PPI in immunology research and this should now be built upon for the advantage of future basic scientific research; PPI can impact greatly on laboratory-based research when given the opportunity to do so.

*PPI is here, ready and waiting to be involved*

– UK-CIC PPI panel

## Supplementary Information

**Additional file 1**: UK-CIC governance structure details and PPI panel profiles.

**Additional file 2**: UK-CIC PPI panel Terms of Reference.

## Data Availability

Not applicable.

## Abbreviations

BSI: British Society for Immunology
HRA: Health Research Authority
NIHR: National Institute for Health Research
PPI: Patient and Public Involvement
UK-CIC: UK Coronavirus Immunology Consortium
UKRI: UK Research and Innovation
