# Supplementary material for: The impact of patient and public involvement on COVID-19 immunology research: experiences from the UK Coronavirus Immunology Consortium
Source: Res Involv Engagem. 2023 May 22;9:34. doi: 10.1186/s40900-023-00446-1 (PMC10201499; doi:10.1186/s40900-023-00446-1)
Supplement: Supplementary file 1 — Additional file 1: UK-CIC governance structure details and PPI panel profiles. [file 40900_2023_446_MOESM1_ESM.docx]

Additional file 1: UK-CIC Governance and PPI panel profiles

**Governance**

The UK-CIC Management Board was chaired by the Principal Investigator, Professor Paul Moss from the University of Birmingham, assisted by co-chair Professor Peter Openshaw (Imperial College London), and comprised five workstream leads. These were responsible for ensuring the consortium delivered on its key aims by leading the research, reviewing progress against milestones, and approving plans and reports.

The Scientific Advisory Board was responsible for providing independent advice to the Principal Investigator and the Management Board of UK-CIC. It acted as a 'sounding board' for the Management Board as research findings progressed and discussed the relevance of emerging global research to the consortium. Its members included world-leading immunologists (independent from the consortium) and two representatives of the Patient and Public Involvement (PPI) panel; this ensured that the priorities for, and impacts of, research on patients and the wider public were considered at all stages of the project. The chair of the Scientific Advisory Board, Professor Arne Akbar (University College London) also sat on the Management Board and ensured the voice of the PPI panel was represented in discussions. The PPI panel worked with the Advisory Board by providing advice, guidance and feedback on the progress of the research and their unique perspectives on the wider implications of findings.

The five workstream leads and individual project leads were invited by the BSI to attend a PPI meeting. The PI and co-chair also suggested which researchers could benefit from meeting the PPI panel. Researchers were told about the PPI element within full consortium meetings and internal communications (e.g. monthly newsletter). The workstream leads sat on the Management Board and they cascaded information about the PPI panel to the research groups within their workstream.

**PPI panel profiles**

Tony Kelly

British born Tony Kelly, Jamaica raised, returned to England in 1979.  Tony worked for 30 years as an Equality & Diversity manager with a Socio-Legal studies Masters Degree from University of Birmingham.


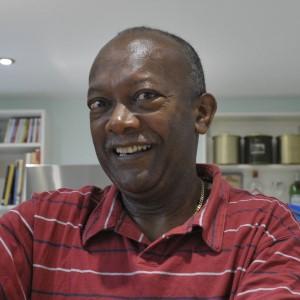
For nearly 8 years was a Diabetes UK Community Champion. His diabetes is controlled by physical activity & diet since diagnosis 17 years ago. Now a Diabetes Strategic Patient Partner - NHS Birmingham &Solihull ICS.

In 2018 Tony delivered diabetes sessions in Dominica for London School of Hygiene &Tropical Medicine/Queen’s Diamond Jubilee Trust.  He is a Personal and Public Involvement representative at three universities.

After his two year personal campaign Public Health England changed the diabetic eye screening test result letters highlighting physical activity.

Tony hosts Birmingham’s BreakTru TV ‘Our Health Is Our Wealth’ and is a health & well-being multi-award winner.

Robert Jasper


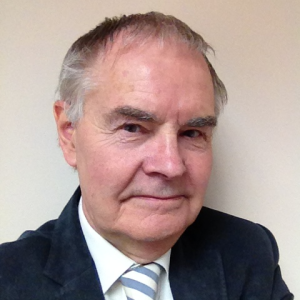
Robert is the founding member of the PPI group at UHB concerned with muscle health, which is an important part of the Birmingham Biomedical Research Centre (BRC) with particular reference to Sarcopenia research.

In this role, he attends the Executive Board meetings of the BRC providing the various research academics and clinicians a public perspective to on their research projects.

Muscle health is vital to keeping the population healthy. Poor muscle health is usually associated with the inactivity due advancing age, after surgery, as a side effect of arthritic conditions and as a result of the modern lifestyles we all lead. It is now an important consequence of “long covid”, hence his interest in the PPI aspect of UK-CIC research.

He also volunteers at his local Stourbridge GP practice (28 GPs and over 30,000 patients) in a Patient Support role. He says this keeps him in touch with “grass roots” patient’s view of the health system.

Debs Smith


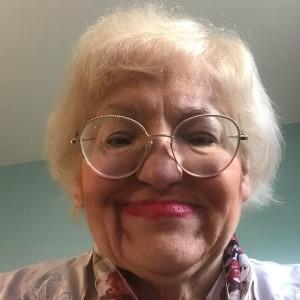
Debs been doing PPI work since 2011. She has been on trial steering and trial management committees for a number of studies. She has worked with many universities from Kent to Glasgow.

Debs also reviews for the National Institute for Health Research (NIHR), the HTA, and the Alzheimer's Society, as well as the Parkinson’s Society. She is also in the British Heart Foundation's PPI group.

 Adrienne Morgan


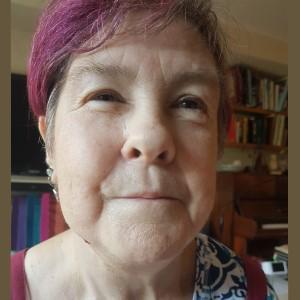
Adrienne was diagnosed with breast cancer in 2005 and metastatic breast cancer in 2010.

After breast cancer she worked in the cancer charity sector, spending three years as the staff scientist at Children with Leukaemia, and then a year as the secretariat for Cancer52 – an umbrella organisation that represents the less common cancers.

She worked as a patient advocate with Breakthrough and Breast Cancer Campaign and now with the new merged charity Breast Cancer Now.  In 2009, with Maggie Wilcox, she was instrumental in establishing the Patient Advocate charity Independent Cancer Patients’ Voice (ICPV) which has grown and is now well respected and much consulted by the cancer trials’ community and has run the world famous Science for Patient Advocates course (VOICE) at Barts.

She is a lay member of several Clinical Trial Management and Working Groups and a familiar independent patient voice in Q and A sessions at cancer research conferences.

Sophia Moreau


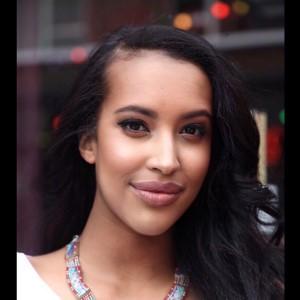
Sophia Moreau is a Corona-survivor and research enthusiast. She has lived experience of invisible disability and is passionate about inclusion. In her day job, Sophia runs the Public Affairs and Policy department of the Small Charities Coalition, representing 91% of charitable groups in the United Kingdom.

Sophia freelances as an opinions writer and investigative journalist, with clients including the BBC, iNews and Huffington Post. Sophia has held advisory positions and acted as a critical friend on various research projects, including the Asian Resource Centre of Croydon's 2020 report on BAME perceptions of COVID-19.

Vivienne Wilkes

**
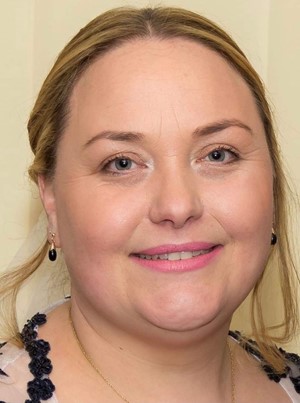
**Vivienne is from East Yorkshire and has been a member of various PPI panels for several years in her local community and on a National level. She has been on panels looking at equality and diversity, internal communications, and research. She has a particular interest in the disability sector both on a personal level and for the wider community with a particular interest in the blind and visually impaired communities.

She feels that PPI gives the opportunity for different perspectives to be heard in order to improve the quality and relevance of research and projects that the PPI is linked to. It is extremely important to her that people who have direct experience of the issues that are for discussion have the opportunity to feed into research and projects that particularly interest or concern them and so that they can help the wider community have a voice through them.

She has a true passion for the role of a PPI panel and hopes that she will play a key role in UK-CIC's PPI panel as it moves forwards in the coming months.

Mo Hafeez

Mo Hafeez is a carer for their young disabled son and lives with multiple long-term health conditions. They offer their experiences to many local communities, NHS services, Universities and PPI panels. They are practiced at analysing research documents and data as well as consulting on projects that work with young people with complex needs. They share knowledge and compassion on projects and ask questions that can make research better and save lives.

Lynn Laidlaw

Hey, my name is Lynn, I live with a rare auto immune disease and multiple long term conditions. I got involved with the UK-CIC PPI panel as I believe involving people with lived experience of conditions is essential to make sure Research is practical and relevant. I am an experienced Public Contributor and work with a number of medical research charities, University PPI groups, organisations such as the HRA and individual researchers. I have experience of reviewing grant applications, sitting on funding sub committee's, reviewing journal articles, a co applicant on research grants and lay member of Trial Management Groups and co author of research articles.  I am a patient representative on a few Research Advisory Groups and have spoken at Conferences and delivered training about Public and Patient Involvement.

Erika Aquino


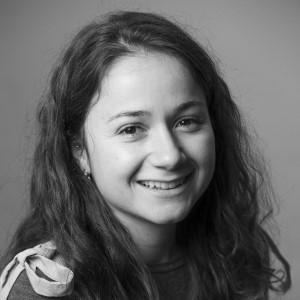
Erika Aquino is the Public Engagement Manager at the British Society for Immunology (BSI) and leads on Patient and Public Involvement (PPI) in UK-CIC. Erika works to engage the public to spark interest in and strengthen understanding of immunology through implementing the BSI’s public engagement strategy. This involves developing and delivering all public engagement activities, supporting BSI members’ involvement in this area, as well creating materials and resources suitable for all.

The current focus of Erika’s work is developing activities to engage with the public about COVID-19 immunology and ensure that the expert scientific voice is heard and reaches a diverse audience. Whilst previously working at Breast Cancer Now, Erika involved patients in co-creating improvements in NHS breast cancer services, collaborating closely with healthcare professionals.

Within UK-CIC, Erika will support the PPI panel members to enable meaningful involvement, co-Chair the meetings to assist the smooth running of the panel and champion the vital role PPI has on the research project.

Other PPI Panel members:

Phil Collis

U Hla Htay
