# Supplementary material for: The impact of patient and public involvement on COVID-19 immunology research: experiences from the UK Coronavirus Immunology Consortium
Source: Res Involv Engagem. 2023 May 22;9:34. doi: 10.1186/s40900-023-00446-1 (PMC10201499; doi:10.1186/s40900-023-00446-1)
Supplement: Supplementary file 2 — Additional file 2: UK-CIC PPI panel Terms of Reference. [file 40900_2023_446_MOESM2_ESM.docx]

## UK Coronavirus Immunology Consortium – Public and Patient Involvement

### **UK-CIC PPI panel**

### **Terms of reference (written September 2020)**

#### Purpose / role of the group:

The panel was established in September 2020 to support the newly formed UK Coronavirus Immunology Consortium (UK-CIC). The aims of the PPI panel will be to advise and guide the UK-CIC Advisory Board through:

- Regularly assessing if the project is on track to answer the research questions ([see summary of research questions](https://www.immunology.org/uk-coronavirus-immunology-consortium-scientific-summary))
- Providing open, honest feedback and reflections on the research priorities
- Providing perspectives and thoughts on wider implications of the research
- Monitoring how the priorities and implications will evolve with other emerging COVID-19 research
- Providing input into UK-CIC’s activities that are aimed at engagement with the wider public

#### Membership:

There will be up to 10 members on the panel, who should ideally remain members for the 10-month duration of the UK-CIC project. Recruitment criteria is based on the UK-CIC public health research priorities e.g. COVID-19 survivors (both those who had mild and more severe cases of the disease), those in shielding groups with long term health conditions, BAME individuals, older individuals, cancer survivors on immunosuppressive therapies, parents with children at high risk, young people. The panel will be balanced with regards to gender. Members should be located around the four nations. Members must have access to the internet and an accessible device in order to attend virtual meetings via Zoom and an email address for communications. Members should remain up to date with COVID-19 current affairs in the UK to be able to provide accurate perspectives on the wider implications of the research.

#### Responsibilities:

Panel members will:

- Attend meetings and read any papers sent in advance
- Play an active, constructive and co-operative role in meetings
- Respond with comments on documents by the requested dates

#### Accountability:

Two representatives from the panel are responsible for reporting back on activities of the group to the UK-CIC Advisory Board.

#### Ways of working:

The panel will meet once a month until the end of the project on 20 August 2021. Panel meetings will be held to ensure that PPI feedback is represented at crucial time points within the scientific reporting structures. The PPI panel will meet in advance of the Advisory Board meetings so representatives can feedback from panel discussions. Ad hoc meetings may be necessary when significant research findings emerge.

Meetings will be held virtually on Zoom with whole group discussions. It is estimated each meeting with last for an average of 1.5 hours and will not exceed 2 hours. Researchers from the UK-CIC will be invited to panel meetings to discuss progress within their research topic. Members of the Advisory Board and Management Board will be invited to specific PPI panel meetings to engage with the panel on certain topics. PPI panel members will also be invited to join specific Management Board meetings to engage directly with the board. The UK-CIC Project Manager and NIHR Clinical Research Network Lead may also attend meetings as observers.

The British Society for Immunology will support panel members in their role and provide secretariat for the panel, organising and attending meetings, circulating papers and sharing information and resources through email. PPI panel members will follow the [BSI code of conduct](https://www.immunology.org/about-us/code-conduct) during their time on the panel and all involved in the panel will maintain confidentiality of the UK-CIC within the project team.

The agenda will be generated by the PPI panel chair(s) and with others as appropriate in collaboration with the Advisory Board chair (Professor Arne Akbar) based on the five research themes. Topics will include panel perspectives on research priorities, questions identified by the Advisory Board that need panel input and the impact of relevant COVID-19 emerging research. The agenda will always include feedback from the two representatives on the last Advisory Board meeting.

Members may be contacted between meetings via email for advice/input should the need arise.

Members will be invited to attend the UK-CIC scientific conference to provide PPI representation.

#### Remuneration/Recognition:

PPI panel members will be remunerated in line with INVOLVE guidelines. Panel members should not be worse off for participating and will be remunerated in recognition for their work and service in the UK-CIC. The honorarium will be £45 per meeting to reflect involvement which equates to an activity that requires some preparation – 1-2-hour video conference, with related papers to read in advance.
